# Eculizumab for pediatric dense deposit disease: A case report and literature review 

**DOI:** 10.5414/CNCS110309

**Published:** 2020-12-10

**Authors:** Katsuaki Kasahara, Yoshimitsu Gotoh, Hisakazu Majima, Asami Takeda, Masashi Mizuno

**Affiliations:** 1 Department of Pediatric Nephrology,; 2 Department of Nephrology, Japanese Red Cross Nagoya Daini Hospital, Nagoya-shi, Aichi-ken, and; 3 Department Renal Replacement Therapy, Division of Nephrology, Nagoya University Graduate School of Medicine, Nagoya-shi, Aichi-ken, Japan

**Keywords:** dense deposit disease, C3 glomerulopathy, eculizumab, C5b-9, alternative complement pathway

## Abstract

Dense deposit disease (DDD), a subtype of complement component 3 (C3) glomerulopathy (C3G), results from alternative complement pathway hyperactivity leading to membrane attack complex formation. DDD treatment strategies are limited. We report a case of a 13-year-old girl diagnosed with DDD at 9 years of age, with nephritic and nephrotic syndrome and C3 nephritic factor-negative alternative complement pathway activation. Initial treatment with prednisolone, methylprednisolone pulses (MPs), and mizoribines was effective for 3 years, after which she relapsed. Despite MP treatment followed by prednisolone and mycophenolate mofetil (MMF), her kidney function and proteinuria deteriorated with a high soluble (s)C5b-9 level; she also developed dyspnea and pleural effusion (PE). Three days after the first eculizumab (ECZ) infusion, urine volume increased, respiratory condition improved, PE resolved, and proteinuria decreased in 1 month. Serum creatinine level decreased, and kidney function completely normalized within 7 weeks. The sC5b-9 level normalized, and although proteinuria decreased, nephrotic range proteinuria persisted during ECZ treatment with MMF for 53 weeks, even with increased treatment interval. Thus, complement activation pathway-targeted therapy may be useful for rapidly progressing DDD. Our data support the role of complement pathway abnormalities in C3G with DDD.

## Introduction 

Dense deposit disease (DDD) is a rare subtype of complement component 3 (C3) glomerulopathy (C3G) defined by the glomerular deposition of C3, but not other immunoglobulins (Igs), and intramembranous electron-dense material in the glomerular basement membrane, caused by the dysregulation of the alternative complement pathway [1]. It mostly affects children, with 59% of patients being < 16 years of age at diagnosis [1, 2]. DDD has a poor prognosis, with more than 70% of affected children developing end-stage kidney disease, and a median onset of 9 years after diagnosis [2]. Treatment strategies for DDD include renin-angiotensin system inhibition, periodic fresh frozen plasma (FFP) infusions, plasma exchange, and corticosteroid, cyclophosphamide, mycophenolate mofetil (MMF), and rituximab administration [3, 4]. Although the Kidney Disease: Improving Global Outcomes (KDIGO) recommended a treatment approach for C3 glomerulopathy in 2017, there are no randomized trials to inform therapeutic decisions, and DDD prognosis remains poor [1, 3, 5, 6, 7, 8]. 

The pathogenesis of DDD offers novel therapeutic opportunities to target complement pathways. Eculizumab (ECZ), a humanized monoclonal antibody against human C5, blocks the activation of the alternative complement pathway and prevents the generation of terminal complement complex C5b-9 [9, 10, 11, 12, 13, 14, 15, 16, 17, 18, 19, 20, 21]. ECZ was initially developed for paroxysmal nocturnal hemoglobinuria [22] but is now widely used for atypical hemolytic uremic syndrome [23] and as an off-label therapy for DDD, with varying results [9, 10, 11, 12, 13, 14, 15, 16, 17, 18, 19, 20, 21]. Here, we report a pediatric patient with proliferative DDD resistant to immunosuppressive therapies who showed a substantial improvement with ECZ treatment. 

## Case report 

**Figure 1.**
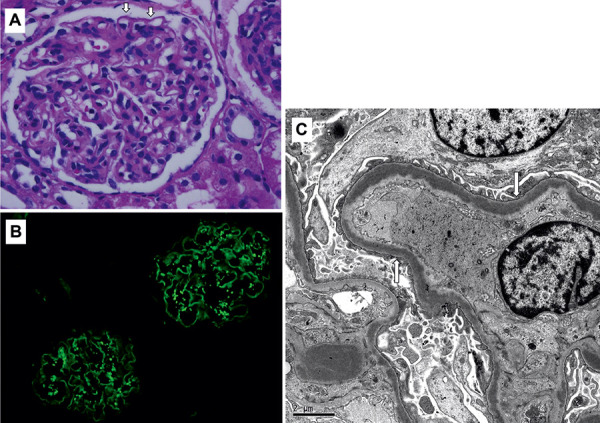
Kidney biopsy: Histological findings in renal biopsy specimens. A: Microscopic features (hematoxylin and eosin staining, × 400): glomeruli show moderate mesangial proliferative lesions, and neutrophils and mononuclear cells are accumulated in the capillary loops. B: Staining of isolated C3 deposition along the glomerular capillaries and mesangium. C: Electron microscopy: the basement membrane shows thickening with dense deposits (arrows).

A 9-year-old female patient presented with macrohematuria and persistent proteinuria (0.2 g/g creatinine) for several months, high serum creatinine level (0.58 mg/dL), low serum creatinine-based estimated glomerular filtration rate (Cr-eGFR) of 81 mL/min/1.73m^2^ based on Uemura’s eGFR formula for Japanese children [24], hyperactivity of the alternative complement pathway (C3 < 10 mg/dL, hemolytic complement (CH50) activity < 12.0/mL), and negative C3 nephritic factor (C3NeF). No pathogenic mutation of the alternative complement genes or abnormalities in C3, factor H, factor I, factor B, membrane cofactor protein, or complement factor H (CFH)-related proteins were observed. Kidney biopsy revealed diffuse endocapillary and mesangioproliferative glomerulonephritis, glomeruli showed moderate mesangial proliferative lesions, and neutrophils and mononuclear cells were accumulated in capillary loops, which were irregularly thickened (Figure 1A). Immunofluorescence showed strong staining of isolated C3 with deposition along the glomerular capillaries and mesangium (Figure 1B). There was no deposition of IgG, IgA, or C1q antibodies. Electron microscopy revealed electron-dense deposits along the glomerular basement membrane (Figure 1C). Therefore, we diagnosed the patient with DDD. 

Methylprednisolone pulses (MPs) were administered at 1 g/day, 3 times a week for 3 weeks, and were followed by prednisolone, mizoribine, dipyridamole, and warfarin therapies. These treatments resulted in complete renal function recovery (serum creatinine level, 0.45 mg/dL; Cr-eGFR, 117 mL/min/1.73m^2^) and normalized urine for 3 years. During this time, C3 and CH50 were undetectable (C3 < 10 mg/dL, CH50 < 12.0/mL). However, when the patient was 13 years old, nephritic and nephrotic syndrome relapsed. A second biopsy confirmed DDD with severe diffuse endocapillary and mesangial proliferative glomerulonephritis with 1 cellular crescent. Treatment with MP, angiotensin-converting enzyme inhibitor (ACEI), and angiotensin II receptor blocker (ARB) was initiated, followed by treatment with prednisolone, dipyridamole, warfarin, and MMF (900 mg/m^2^) instead of mizoribine, for 1 month. However, her kidney function further deteriorated, with Cr 1.02 mg/dL (Cr-eGFR 57 mL/min/1.73m^2^), proteinuria increased (12.3 g/g creatinine), and dyspnea requiring oxygen occurred owing to pleural effusion (PE). Although FFP was administered, there was no change. 

**Figure 2.**
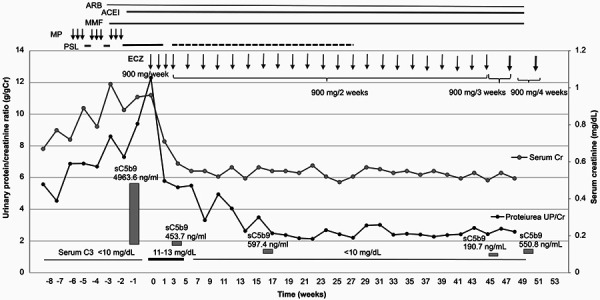
Clinical course and medication of the patient. PSL = prednisolone; MMF = mycophenolate mofetil; ECZ = eculizumab; MP = methylprednisolone pulses; ACEI = angiotensin-converting enzyme inhibitor; ARB = angiotensin II receptor blocker; Cr = creatinine; UP/Cr = urinary protein/creatinine ratio.

Following anti-Neisseria meningitis immunization, ECZ treatment was initiated at 900 mg/week for 4 weeks, and then the same dose was administered every 2 weeks until week 49 (Figure 2). The day after the first ECZ infusion, leukocyturia disappeared. Three days after the first ECZ infusion, the serum creatinine level began to decrease (from 0.96 to 0.78 mg/dL), and the kidney function completely normalized within 7 weeks (Figure 2). Her urine volume increased, and as respiration recovered, oxygen therapy was discontinued. Although elevated soluble (s)C5b-9 level (4,963.6 ng/mL; normal range 148.0 – 1,243.6 ng/mL [25]) was recorded during the acute phase of DDD, it decreased to 453.7 ng/mL 3 days after the first ECZ infusion. Immediately before the 10^th^ ECZ infusion, the sC5b-9 level was within the normal level (597.7 ng/mL) (Figure 2). In the long term, PE resolved, and proteinuria decreased from 12.9 to 2.5 g/g creatinine within 1 month (Figure 2). No adverse effects were observed. 

After discharge from the hospital, treatment was continued in an outpatient ward with MMF, ARB, ACEI, dipyridamole, and gradual prednisolone tapering. At the third biopsy after the 41^st^ ECZ infusion, immunofluorescence indicated that isolated C3 deposits along the glomerular basement membrane and IgG deposition had decreased compared with those at the second biopsy. 49 weeks after the first ECZ treatment, the treatment interval was extended from 2 to 3 or 4 weeks. Under this regimen, the sC5b-9 level was normal (190.7 and 550.0 ng/mL at 3 and 4 weeks, respectively), and kidney function (Cr-eGFR 107 mL/min/1.73m^2^), proteinuria (~ 2.5 g/g creatinine), and hematuria (10 – 19/HPF) were stable throughout the 53-week follow-up period; leukocyturia was not observed. After the 53-week follow-up period, proteinuria and leukocyturia started to increase at 6 weeks after the final ECZ infusion. At 17 weeks after the final ECZ infusion, nephritic and nephrotic syndromes reappeared, and we restarted ECZ treatment. Consequently, leukocyturia disappeared and proteinuria started to decrease at 1 week after ECZ retreatment without kidney injury. 

## Discussion 

We observed an impressive and significant response to ECZ in a pediatric patient with DDD, despite the lack of a notable response to other immunosuppressive therapies. Proteinuria decreased over the 53-week treatment period, even when the treatment interval was extended from 2 to 3 or 4 weeks (Figure 2). 

DDD treatment has historically focused on supportive measures (ARB and ACEI), plasmapheresis to remove autoantibodies, and antiproliferative agents such as prednisolone, MMF, and anti-CD20 antibody [3, 4, 5, 6, 7, 26]. We also used treatments based on IgA nephropathy such as dipyridamole, warfarin, and both ARB and ACEI [27, 28]. KDIGO recommends the treatment of moderate C3G disease with prednisone and MMF [8]. ECZ has shown efficacy in a small number of C3G patients with DDD, and the KDIGO recommends the use of ECZ for severe disease [8, 9, 10, 11, 12, 13, 14, 15, 16, 17, 18, 19, 20, 21]. ECZ is a humanized monoclonal antibody that binds to C5 with a high affinity, thereby blocking the terminal complement complex and preventing membrane attack complex formation. Data from several case reports and series suggest that some [9, 10, 11, 12, 13, 14, 15, 16, 17, 18, 19], but not all [20, 21], patients with DDD (including C3G) may benefit from ECZ. The variability in response could be related to the extent of terminal complement activation, which can differ substantially among patients [9, 10, 11, 12, 13, 14, 15, 16, 17, 18, 19, 20, 21]. However, we believe that a high sC5b-9 level is an important determinant [11], and elevated sC5b-9 level before treatment may help predict treatment response [12]. Most patients with no response or weak responses to ECZ do not show preceding high sC5b-9 level or do not have their sC5b-9 level measured before treatment [9, 10, 11, 12, 13, 14, 15, 16, 17, 18, 19, 20, 21]. In one study, ECZ treatment completely normalized the extremely high sC5b-9 level (> 1,000 ng/mL) in patients and significantly decreased proteinuria (p = 0.006) [11]. There have been no reported adverse effects of ECZ therapy [9, 10, 11, 12, 13, 14, 15, 16, 17, 18, 19, 20, 21]. 

In our patient, at the third biopsy after ECZ treatment accompanied by a substantial decrease in the sC5b-9 level, immunofluorescence of C3 deposits showed an improvement, indicating slowed disease progression [16]. ECZ is a potentially effective treatment for patients with crescentic rapidly progressive C3G [19]. In our patient, a second biopsy indicated mesangial proliferative glomerulonephritis with a cellular crescent before ECZ treatment. 

Use of ECZ for pediatric DDD has been reported in 9 patients (Table 1) [9, 10, 15, 19]. Most children with DDD showed increased C3NeF and/or mutations of the alternative pathway [9, 10, 15, 19]. As detectable C3NeF appears to be a risk factor for a poor response to ECZ [11], our patient, who was C3NeF negative and showed no genetic abnormalities of the alternative pathway, responded well to ECZ. After ECZ treatment, proteinuria decreased in all 9 patients resistant to immunosuppressant therapies, and all except 1 showed improved kidney function [9]; the 1 patient showed decreased kidney function necessitating dialysis before ECZ use [9]. Oosterveld et al. [10] have shown that leukocyturia disappears almost completely within 1 week after the first dose of ECZ. Even in our patient, leukocyturia disappeared the day after the initiation of ECZ treatment, with decreased proteinuria. Thus, leukocyturia might reflect the involvement of the tubulointerstitial apparatus and might be a suitable indicator of disease activity and reaction to therapy [10]. 

To the best of our knowledge, this is the first report of a pediatric DDD patient with information on sC5b-9 level before and after ECZ treatment and treatment interval changes. After a 12-week washout, sC5b-9 should return to levels observed before the first ECZ infusion [11], but this parameter has not been evaluated to date. In our patient, the sC5b-9 level remained normal at 3- to 4-week treatment intervals, and kidney function, proteinuria, and hematuria remained stable. Because proteinuria and leukocyturia started to increase 6 weeks after the final ECZ treatment without kidney injury, we retreated the patient with ECZ at 17 weeks after the final ECZ treatment, and proteinuria and leukocyturia decreased immediately. Thus, if the sC5b-9 level cannot be measured, proteinuria and leukocyturia might be indicators for the use of ECZ. 

The contribution of combined immunosuppressive therapies to our patient’s clinical response cannot be ruled out. However, these therapies had been applied for more than 1 month before ECZ treatment, with continued deterioration of kidney function and proteinuria. In contrast, an improvement was evident within days after the first ECZ treatment, with further biopsy and sC5b-9 measurement showing continued resolution. This response is similar to that reported in previous cases [11, 18], suggesting that ECZ was responsible for the improvement in this case. 

Interestingly, the ECZ dose used in this study was different from the recommended dose. For patients with a body weight over 40 kg, the normal practice is to administer ECZ at 900 mg/week for the first 4 weeks and then use 1,200 mg/week in the subsequent weeks [22, 23]. However, the weight of our patient decreased to < 40 kg in week 5, after the fourth dose of ECZ; therefore, we used 900 mg/week, instead of 1,200 mg/week, from week 5 onward. 

In conclusion, we report a case of a pediatric patient with proliferative DDD resistant to immunosuppressive therapies, who received ECZ for 53 weeks. Importantly, her condition and sC5b-9 level were stable even with prolonged treatment interval. Our findings contribute to the pool of evidence supporting the pivotal role of complement alternative pathway abnormalities in DDD. 

## Acknowledgment 

The authors thank Dr. Inoue, Dr. Hidaka, Dr. Fukumori, and the Japanese Association for Complement Research for genetic analysis. 

## Funding 

The Japanese Association for Complement Research for Genetic Analysis, which was supported by Alexion Pharma GK as a company-sponsored research grant. 

## Conflict of interest 

All authors have no conflict of interest to declare. 

**Table 1.** Summary of clinical and laboratory features of pediatric dense deposit disease.

	Our case	Rousset-Rouviere et al. 2014 [15]	Ozkaya et al. 2014 [19]	Oosterveld et al. 2015 [10]	Holle et al. 2018 [9]
Total	1	1	1	5	2
Sex (male/female)	1/0	1/0	0/1	2/3	ND
Age at diagnosis (years)	9	8	14	8.4 (5.9 – 13)	(No 1) 2, (No 2) 13
Nephritic and/or nephrotic syndrome	1/1	1/0	0/1	5/3	ND
Low circulating C3 (g/L)	Yes (< 0.1)	Yes (< 0.04)	Yes (0.16)	Yes (0.28: 0.05 – 0.57)	Yes (No 1: 0.09, No 2: 0.23)
sC5b-9 levels before ECZ (ng/mL)	4,963.6	ND	ND	1,238 (3.47 – 2,467)	No 1: ND, No 2: 1,107
sC5b-9 levels after ECZ (ng/mL)	550	ND	ND	ND	No 1: 157, No 2:104
Increased C3NeF	No	Yes	Yes	Yes (3)	Yes (1)
Genetic analysis of the alternative pathway	Negative	Normal factor H, I, and membrane factor of proteolysis	CFH gene: polymorphism (p).V62I, p.H402Y	3 positive (deletion for CFHR1-CFHR3)	p.H402Y, p.A307A+, p.A473A, p.W32R, pV62I, p.Q672Q, p.E936D
ECZ duration (weeks)	52	28	31	80 (64 – 112)	ND
Prior ECZ therapy	MP, PSL, MMF, ARB, ACEI, FFP	Solu-Medrol, CSL, MMF, ACEI, RTX	MP, PSL, CPA, ACEI, ARB, PE, FFP	MP (3), PE (3), PSL (3), CsA (1)	Steroids (2), MMF (2), RTX (2), PE (2), TCL (1), ACEI (2)
eGFR before ECZ (mL/min/1.73 m^2^)	57	(serum Cr 5.5 mg/dL)	(serum Cr 0.49 mg/dL)	59 (23 – 93)	No 1: 91.0, No 2: 137.7
eGFR after ECZ (mL/min/1.73 m^2^)	107	(serum Cr 0.9 mg/dL)	(serum Cr 0.5 – 0.6 mg/dL)	94 (68 – 101)	No 1: 47.3, No 2: 128
Maximum urinary protein/creatinine ratio before ECZ (g/g Cr)	12.9	~ 2.5	(24 h urine protein excretion: ~ 10 g/day)	9.6 (7.7 – 14.8)	No 1: 1.9, No 2: 2.5
Urinary protein/creatinine ratio after ECZ (g/g Cr)	2.5	(< 0.5 g/day)	(< 0.2 g/day; after 31 weeks of ECZ)	0.51 (0.37 – 0.62)	No 1: 0.1, No 2: 0
Time to complete remission (weeks)	No remission	12	31	ND (improved significantly within 12 weeks)	ND

MMF = mycophenolate mofetil; RTX = rituximab; ACEI = angiotensin-converting enzyme inhibitor; ARB = angiotensin II receptor blocker; PSL = prednisolone; CsA = cyclosporine; CPA = cyclophosphamide; mPSL = methylprednisolone; CSL = corticosteroid; TCL = tacrolimus; MP = methylprednisolone pulses; PE = plasma exchange; ND = no data; ECZ = eculizumab; IVIG = intravenous immunoglobulin; sC5b-9 = soluble C5b-9; eGFR = estimated glomerular filtration rate. Nephrotic syndrome = severe proteinuria (> 3.5 g/1.73 m2) or presence of proteinuria (≥ 40 mg/m^2^/h) and serum albumin level < 25 g/dL. Nephritic syndrome = proteinuria + hematuria + hypertension ± renal failure. Renal failure = GFR < 90 mL/min/1.73m^2^. Median (interquartile range).
